# Oral Mucosa, Saliva, and COVID-19 Infection in Oral Health Care

**DOI:** 10.3389/fmed.2021.656926

**Published:** 2021-04-22

**Authors:** Devi Sewvandini Atukorallaya, Ravindra K. Ratnayake

**Affiliations:** Department of Oral Biology, Dr. Gerald Niznick College of Dentistry, Rady Faculty of Health Sciences, University of Manitoba, Winnipeg, MB, Canada

**Keywords:** oral epithelial cells, saliva, taste, SARS-CoV-2, ACE2 receptor, COVID-19

## Abstract

The SARS-CoV-2 virus has shaken the globe with an ongoing pandemic of COVID-19 and has set challenges to every corner of the modern health care setting. The oral mucosa and saliva are high risk sites for higher viral loads and dental health care professionals are considered a high risk group. COVID-19-induced oral lesions and loss of taste and smell are common clinical complaints in the dental health care setting. The SARS-CoV-2 virus has been found to cause a wide range of non-specific oral mucosal lesions, but the specific diagnosis of these mucocutaneous lesions as COVID-19 lesions will facilitate the prevention of SARS-CoV-2 in dental health care settings and aid in proper patient management. The reported loss of taste and smell needs further investigation at the receptor level as it will give new insights into SARS-CoV-2 pathogenicity. The high yield of virus in the salivary secretion is a common finding in this infection and ongoing research is focusing on developing saliva as a rapid diagnostic fluid in COVID-19. In this review, we discuss the significance of oral mucosa, saliva and the relevance of the COVID-19 pandemic in dentistry.

## Introduction

Coronavirus disease 2019 (COVID-19) is an infectious disease that was first detected in large numbers in Wuhan, China; it is caused by a newly discovered coronavirus identified as severe acute respiratory syndrome coronavirus 2 (SARS-CoV-2) (1). Coronaviruses are large RNA viruses with beta coronaviruses, including SARS-CoV and SARS-CoV-2, having been shown to be the deadliest viruses, causing respiratory distress syndrome (2, 3). Since 1960, six coronaviruses have been found to cause diseases in humans. In 2002, SARS-CoV caused a major outbreak known as severe acute respiratory syndrome (SARS), which caused about 10,000 fatalities worldwide (4). Only a decade later, another pathogenic coronavirus, known as the Middle East respiratory syndrome coronavirus (MERS-CoV), caused an endemic in Middle Eastern countries (4, 5). SARS-CoV-2 is the seventh member of the coronavirus family to affect humans (4). Interestingly, the genome of SARS-CoV-2 aligned with the genomes of viruses from bats (Bat-CoV and Bat-CoV RaTG13) in *Rhinolophus affinis* species of the Yunnan province with 96% similarity; structural analysis revealed a mutation in the envelope protein (Spike protein) and nucleocapsid protein (6). The coronavirus has a simple structure with few proteins (7). There are 4 major structural proteins: the envelope protein (E), spike protein (S), transmembrane protein (M), and nucleoprotein (N). The E, S, and M proteins facilitate virus entry into the host cells, virion assembly, and viral pathogenesis. The viral genome, is in close association with N protein and also aid the E protein in virion assembly (7).

At present, two modes of transmission for SARS-CoV-2 have been identified: direct and indirect transmission. Direct transmission includes contact with the infected individual's body fluids, respiratory or salivary droplets and, other body fluids such as feces, urine, semen, and tears (8). The signs and symptoms of COVID-19 can be divided into respiratory and extra-respiratory manifestations. The most common reported respiratory signs are cough, fever, and dyspnoea (9–11). There is a wide range of extra-respiratory signs and symptoms, including oral mucosal lesions and neurological dysfunctions, such as loss of smell, loss of taste, headache, and associated myofascial pain; these are now included in the diagnostic criteria of this disease (Table 1) (10, 19).

**Table 1:** Orofacial manifestations of COVID-19.

**Clinical features**	**References**
Headache	(12–15)
Myofacial pain	(13–15)
Oral ulcerations	(14–16)
Burning sensation of the oral mucosa	(14, 16)
Oral vesicle formation	(16, 17)
Loss of taste	(14–16, 18)
Loss of smell	(14–16, 18)
Dry mouth	(14, 15)
Skin discomfort	(16)

The nasal cavity, nasopharynx, oropharynx and oral cavity are identified as potential replication sites for the SARS-CoV-2 virus (20, 21). The oral cavity, which is rich in saliva and the oral microbiome, is a well-known site harboring various types of respiratory viruses (22, 23). The oral saliva has been found to contain a high yield of viruses, suggesting salivary glands as active proliferating sites for this virus (20, 24). Moreover, xerostomia and loss of taste can be associated with the salivary gland dysfunction associated with COVID-19 (23, 25). However, these signs are often masked by the more life threatening respiratory signs and symptoms, which need emergency medical attention most of the time. This review aimed to provide histological specifications of the oral mucosa and its functional significance in SARS-CoV-2 infection, highlighting the orofacial manifestations and its impacts on the dental health profession.

## Oral Mucosa

**Figure 1:**
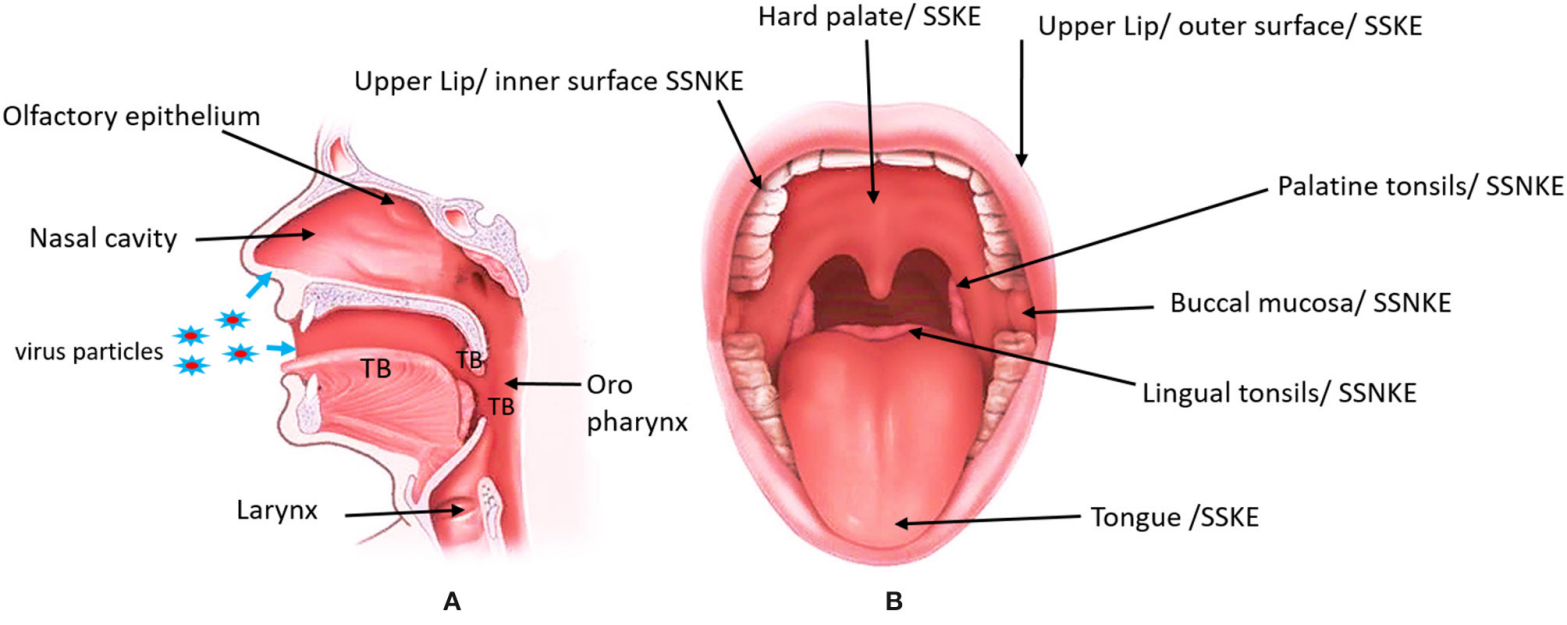
Modified image indicating the location of the entry points of SARS-CoV-2 and the anterior view of the oral cavity labeling different areas of the oral mucosa. **(A)** Blue arrows indicate the nasal and oral entrance of the virus. The location of the olfactory epithelium and taste buds (TB). Olfactory epithelium is located on the roof of the nasal cavity. Taste buds can be found in the tongue, tonsils and oropharynx. **(B)** The specific location of the Stratified squamous keratinized epithelium (SSKE) and Stratified squamous non keratinized epithelium (SSNKE) in the oral cavity. https://www.informedhealth.org/how-do-the-tonsils-work.html, “*How do the tonsils work?”* Institute for Quality and Efficiency in Health Care (IQWiG, Germany), 17 Jan 2019.

The oral mucosa is the specialized mucous outer covering layer of the oral cavity which consists of the stratified squamous epithelium and the underlying connective tissue (lamina propria) (Figure 1) (26). Apart from the common epithelial functions, such as protection and lining, oral mucosa is regionally specialized to form special functions like taste perception, sensory perception, mastication, and secretion (26). The oral epithelial cells have numerous structural and functional specifications to withstand physical and chemical attacks. Squamous epithelia possess structural properties like stratification and cornification of the keratinocytes and specific cell-to-cell interactions to maintain their barrier functions (26). The epithelial cells are metabolically active and are capable of reacting to external stimuli by synthesizing a number of cytokines, adhesion molecules, growth factors, and chemokines (27). The oral cavity is a dynamic ecosystem that varies over time in ways that influence spatial patterns of microbial community assembly (28). Among the oral microbial community are common commensals that can be opportunistic pathogens when the host immunity is compromised. There are many species of bacteria, fungi and viruses which are either pathogenic or opportunistic, causing common oral diseases such as caries and periodontitis, oral candidiasis and viral mucosal infections (29, 30).

## Pathogenesis of SARS-CoV-2 in Oral Mucosa

Oral viral infections are a common clinical complaint in dentistry, which is often associated with oral mucosal lesions. The herpes virus group (herpes simplex 1–8), human immune deficiency virus (HIV) and Zika virus are capable of infecting and replicating in the oral mucosa, leading to painful oral ulcers (22, 27). Viruses like paramyxovirus, HIV, cytomegalovirus and Epstein-Barr virus (EBV) have been found to replicate in salivary glands and negatively affect the normal functioning of the salivary glands (22). Several recent reports have described the oral manifestations of SARS-CoV-2 infection such as vesicular bullous lesions and ulceration (24, 25, 31).

The single cell RNA-seq (scRNA-Seq) studies of ACE2 expression have detected high levels of expression in keratinized epithelial cell surfaces of the oral cavity, such as the dorsum of the tongue and hard palate, rather than buccal or gingival tissues (32). In the human body, the ACE2 receptor is known to be important in regulating blood pressure homeostasis by regulating the renin–angiotensin–aldosterone system (RAAS), where it converts angiotensin I to angiotensin II; this cascades body functions to maintain blood pressure and sodium water retention (33). SARS-CoV-2 enters a host's body and invades host cells via the ACE2 membrane receptor; this binding leads to conformational changes and cleavage of the S protein from the virion, and releases the nucleocapsid into the cytoplasm (7, 34). The S protein is proteolytically cleaved by cellular cathepsin L and the transmembrane protease serine 2 (TMPRSS2) (33). Haga et al. found that SARS-CoV viruses can induce tissue necrosis factor (TNF)-α-converting enzyme (TACE)-dependent shedding of the ectodomain of ACE2, and that process was coupled with TNF-α production (35, 36). TNF-α is an inflammatory cytokine produced by macrophages/monocytes during acute inflammation and is responsible for a diverse range of signaling events within cells, leading to cell necrosis or apoptosis (37). These data suggest that cellular signals triggered by the interaction of SARS-CoV with ACE2 are positively involved in viral entry but lead to tissue damage. The presence of high ACE2 expression in the alveolar tissues, oropharyngeal mucosal cells, gastrointestinal tract, kidneys and endothelial cells, including oral tissues, indicated that those organs with high ACE2-expressing cells should be considered to be potential high risk sites for SARS-CoV-2 (21, 32).

## Oral Manifestation of COVID-19

The pathology of viral infections is often associated with either cellular destruction due to viral invasion or the consequence of host immune reaction to the viral antigen (23). In the oral mucosa, viral infections disrupt epithelial cells and trigger local inflammatory reactions which typically present with abrupt onset and the association of solitary or multiple blisters or ulcerations (23). Oral vesicles, blisters, macular popular rashes and ulcerations are the common clinical features of viral infections (23). In SARS-CoV-2, epithelial injury causes similar pathogenic features in the oral tissues, such as ulcers, erosions, bullae, vesicles, pustules, fissured or depapillated tongue, macule, papule, plaque, pigmentation, halitosis, whitish areas, haemorrhagic crust, necrosis, petechiae, swelling, erythema, Kawasaki-like angular cheilitis, atypical Sweet syndrome, and Melkerson-Rosenthal syndrome (19, 25, 38). The most common sites of involvement are the tongue (38%), labial mucosa (26%), and palate (22%) (19, 39). Oral lesions were almost equal in both genders (49% female and 51% male). Patients with an older age and higher severity of COVID-19 disease had more widespread and severe oral lesions (25).

The histological analysis of oral SARS-CoV-2 lesions is associated with defects in the vascular arrangement of the oral mucosa (40). Pathogenesis of oral mucosal lesion of COVID-19 are associated with the accumulation of lymphocytes and Langerhans cells in the vasculature of the subcutaneous junctions and virus induce keratinocyte destruction by the cytotoxic lymphocytes (41). Histological examination of biopsies of COVID-19 patients who also had skin manifestations confirmed the vascular ectasia with dilated capillaries, large blood filled spaces and perivascular lymphocytic infiltrate with eosinophilia (40).

A lack of oral hygiene, opportunistic infections, stress, immunosuppression, vasculitis, and hyper-inflammatory response secondary to COVID-19 were found to be the predisposing factors for the onset of oral lesions in COVID-19 patients (19, 39). Stress-induced oral ulceration can be increased among patients due to the unknown fear of the pandemic. It has been already reported that this pandemic has severely affected the mental health of the global community (42). Patients have reported changes in sensation in the tongue, plaque-like changes in the tongue and swelling in the palate, tongue and gums (25). Tongue lesions may be associated with the increasing activity of viral events on the epithelial mucosa of the tongue (39). On the other hand, immune suppression can lead to the harboring of opportunistic pathogens like *Candida albicans*, which can lead to the above observed tongue lesions (19). SARS- CoV-2 oral lesions healed between 3 and 28 days after they appeared. COVID-19-induced oro-mucosal lesions can be treated with mouthwashes, topical or systemic corticosteroids, systemic antibiotics and antivirals (39, 40). Increasing evidences are suggested that the antiseptic mouthwashes such as chlorhexidine, sodium hypochlorite and povidone-iodine found to be effective in reducing the SARS- CoV-2 viral load in the oral cavity and can be prescribed to patients with mucosal lesions as first line of therapy (43, 44). The topical or systemic corticosteroids, systemic antibacterial and antiviral needs to be prescribed according to the individual patient needs. Multidisciplinary team approach is important when prescribing or continuing systemic corticosteroids, antibiotics or antivirals to COVID-19-induced oro-mucosal lesions (39).

## COVID-19 Induced Taste and Smell Loss

Taste is a special sensation of the human oral mucosa which plays a vital role in the identification of nutrients and regulation of food intake. Humans are capable of detecting five basic tastes: sweet, sour, salt, bitter and umami. Tastes stimulate specialized cells known as taste receptor cells (TRCs), which contain taste signal transduction proteins. Sour and salty tastes modulate the function of TRCs by the direct activation of specialized membrane channels (45, 46). In contrast, sweet, bitter and umami taste transduction is mediated through the G protein-coupled receptor (GPCR) signaling pathway (47). TRCs are locally organized as taste buds (TBs) which are located in the dorsum of the tongue and extra oral taste buds can be found in the tonsils and oropharynx (Figure 1A). TBs are made of receptor cells, support cells and are innervated by branches of the VII (facial), IX (glossopharyngeal), and X (vagal) cranial nerves. Taste information is relayed to the brain and its recognition elicits behavioral responses to the food (48, 49). True loss of taste is extremely rare, and it is usually preceded by the inability to perceive the odor of food due to olfactory dysfunction or the deficiency of saliva to dissolve food molecules to get into the taste receptors (25, 50).

Smells or odorants reach the olfactory epithelium, which covers the cribiform plate and the upper part of the nasal septum and the middle/upper turbinates and dissolve in the mucus layer, binding/activating olfactory receptors (Figure 1A). Up to 30 million receptor neurons, which express up to 350 different olfactory receptors, can be found in the olfactory epithelium. A complex combinatorial coding, by which each odorant ligand may be recognized by an olfactory receptor combination, enables humans to detect billions of different odors. Olfactory information, which is processed and integrated in the olfactory bulb, is then projected onto the primary olfactory centers such as the limbic system (emotions) and the hypothalamus (memory), and is finally projected to the olfactory cortex, where humans acquire the consciousness of smelling (50, 51). Smell loss in respiratory infections are multifactorial and are caused by a combination of the mechanical obstruction of odorant transmission in the olfactory cleft due to mucosal type 2 inflammation (oedema or nasal polyps), leading to shedding, and/or degeneration of the olfactory epithelium and the reduction or loss of the sense of smell (51).

The SARS- CoV-2 infection associated sudden loss of taste and smell was reported in several countries in early March, with the rapid increase in COVID-19 patient numbers. Interestingly, a series of sporadic cases, predominantly in health care workers, reporting a sudden, severe, and sometimes isolated loss of smell and/or taste was reported in different countries (50, 52). Nasal congestion was found to be the driving factor for the loss of smell. It is possible that damage to the olfactory neuroepithelium can cause defects in smell detection. Loss of smell is common among females and loss of smell is associated with a loss of taste most of the time (51, 53).

Still, there are a lack of data on a specific loss of different tastants (flavors) (54). In a web-based questionnaire study (*n* = 128), 67 patients (52%) reported changes in taste sensation. Fifty-two patients reported a change in their spicy taste perception, 54 in salty taste, 53 in sour taste, and 61 in sweet taste. In a comparison between men and women, COVID-19 induce taste changes and changes in taste subgroups were found to be common among women, but this needs further investigations (55, 56). A possible reason for the loss of taste in COVID-19 might be due to the increasing number of ACE-2 receptors on the tongue keratinocytes and the keratinocyte cell death and slough production can block taste buds which can adversely affect taste perception (53, 57). However, the presence of ACE-2 receptor activity on taste receptor cells is unknown at present, hence the specific role of SARS-CoV-2 on specific taste bud cells (receptor cells and supportive cells) needs to be further investigated (57). It has been shown that GPCR can be found in a diverse range of body tissues, not only in the oral cavity but in the lung epithelial cells, blood brain barrier and blood vessels (58). It will be interesting to see the specific role of SARS-CoV-2 and GPCR interactions in terms of COVID-19 pathogenesis. On the other hand, COVID-19 induces salivary gland dysfunction, which leads to dry mouth, and can result in the malfunctioning of taste perception (59). Treatment with artificial saliva can improve the xerostomia-induced taste loss (60). Quantitative smell testing demonstrates that decreased smell function is a major marker of SARS-CoV-2 infection, and suggests the possibility that smell testing may help, in some cases, to identify COVID-19 patients in need of early treatment or quarantine (61). Song et al. found that a loss of taste was more frequent (21%) than a loss of smell (11%) in hospitalized patients, with the loss of taste but not smell being associated with severe COVID-19 (62). Most patients recovered their smell and taste dysfunctions within 2 weeks (50, 62).

Overall, there is no real evidence for any specific pharmacological option for the post-viral loss of smell including COVID-19. Some studies report an improvement in olfactory function following topical or systemic corticosteroid therapy (50, 63). Olfactory training is the only current evidence-based therapeutic option for post-viral olfactory loss, with COVID-19 positive patients reporting an improvement in smell (45.6%) and taste (46.1%) at the time of the survey; in 90.6%, this was within 2 weeks of infection (64). Over 90% of COVID-19 patients with a loss of smell may recover that sense within the first month, and olfactory training is strongly recommended if smell has not recovered after that period of time, but can be started earlier (65).

## Role of Saliva in COVID-19 Pathogenicity and Disease Diagnosis

Human saliva is a unique body fluid of the oral cavity. It is a hypotonic solution of salivary acini, gingival crevicular fluid and oral mucosal exudates (66). Approximately 90% of saliva is secreted from the salivary glands; the major glands include the parotid glands, submandibular glands and sublingual glands (66). The salivary glands are highly vascular structures, where there is a constant exchange of substances. A normal person produces 600 mL of saliva per day. It is mainly composed of water (94–99%), with organic molecules accounting for ~0.5% and inorganic molecules for 0.2% (66). It has the functions of lubricating the oral mucosa, digesting food, and cleaning and protecting the oral cavity, and is one of the most important factors affecting homeostasis of the oral cavity.

Viral infections are often associated with the infection induced inflammation of the salivary gland (22). Saliva based biomarkers are useful in diagnosis of several viral infections such as hepatitis A virus, hepatitis B virus, hepatitis C virus, HIV-1, measles virus, rubella virus, and mumps virus (66). Several routes of SARS-CoV-2 viral entry into the saliva have being suggested. There is direct entry to the oral cavity from upper and lower respiratory tract secretions, while circulatory viruses in the blood enter the gingival crevicular fluid. Studies reported a high yield of virus particles in the gingival sulcus and crevicular fluid and are suspected to provide favorable conditions for virus replication and maintenance (32). Moreover, SARS-CoV-2 salivary gland infections can produce large amounts of viruses in the salivary gland tissues and release them into the secretions (67). Studies performed on rhesus macaques found that there is a rapid infection in the salivary gland epithelial cells by SARS-CoV, suggesting salivary glands as very early proliferating sites for coronaviruses (68). Hence, increased ACE-2 expression in minor salivary glands compared to the lungs suggestive of salivary glands as an early target organ and saliva can be a vital source in the early diagnosis of disease before the respiratory symptoms appear (20, 31, 68).

In COVID-19, the impaired salivary gland secretions are often associated with xerostomia and taste loss (69). Xerostomia is the subjective complaint of oral dryness, while salivary gland hypofunction is an objective matter characterized by reduced salivary flow (70, 71). In SARS-CoV infections, xerostomia could be aggravated by impaired nasal breathing due to nasal congestion and rhinorrhea, where the oral breathing increase and it can impaired salivary gland function and xerostomia is secondary (25). Similar to COVID-19-induced oral mucosal lesions, pandemic-induced psychosocial factors have a greater impact on normal salivary gland function and quantitative secretions (25, 59).

The saliva-based COVID-19 diagnosis is getting increased attention for several important reasons. First, saliva specimens can be easily obtained, by asking patents to spit into a container, which is not an invasive procedure and minimizes the chance of exposing health care workers to the highly infectious SARS-CoV-2 virus; it is also ideal for testing the elderly vulnerable population, pediatric patients and community settings, where large sample collection is needed (Table 2) (87). There is a 92% positive rate of SARS-CoV-2 in saliva compared to nasopharyngeal aspirate and live virus can be successfully cultivated through saliva samples, highlighting the value of saliva in the diagnosis of COVID-19 (88). As discussed previously, the early detection of SARS-CoV-2 in the saliva can be vital in diagnosing COVID-19 patients before respiratory symptoms appear, which greatly aids in controlling public health measures such as the quarantine process (20, 88, 89).

**Table 2:** Articles related to COVID-19 and dentistry.

**Country**	**Study overview**	**Article type**	**References**
Italy	Prevention of spread of SARS-CO-V-2	Review	(72)
Italy	Symptoms/signs, protective measures, awareness, and perception levels regarding COVID-19 among dentists in Lombardy, Italy	Questionnaire survey	(73)
China	COVID-19 and management protocols for dental practitioners and students	Review	(74)
Italy	Prevention of COVID-19 in Pediatric dentistry	Review	(75)
Brazil	Oral Manifestations in Patients with COVID-19	A living systematic review	(76)
Italy	Infection control in Dentistry	Review	(77)
Italy	Oral manifestations of COVID-19	A narrative review	(78)
Spain	Oral lesions of COVID-19	Cross sectional study	(79)
Multicenter study	Endodontic emergency management by endodontists and general dental practitioners in COVID-19 times	Online survey using questionnaire	(80)
USA	Epidemiology, symptoms, and routes of transmission of COVID-19	Review	(81)
India	Safety operative protocols	Commentary	(82)
France	Salivary and Nasal Detection of the SARS-CoV-2 Virus After Antiviral Mouth rinses	Randomized control trial	(83)
Brazil	Mouth wash reducing viral load of COVID-19	Systematic review	(84)
Nigeria	Impact on orthodontic patients	Online questionnaire cross-sectional descriptive study	(85)
India	Appropriate orthodontic appliances during the COVID-19 pandemic	Scoping review	(86)

## Future Perspectives With Regard to the Oral Health Profession

On March 11th 2020, the World Health Organization (WHO) declared COVID-19 to be a global pandemic. As of the 20th January 2021, 96,866,468 cases had been reported globally, with 20,72,466 deaths. The first cases of COVID-19 were seen in Canada on February 10th 2020, and there have since been 723,908 cases and 18,421 deaths (90). The current public health regulations to prevent the spread of this virus have been based on the modes of transmission. Following the strict global (WHO) and Canadian public health guidelines was found to be effective in preventing the spread of COVID-19 (91, 92). Several excellent research and review articles have already been published on the impact of COVID-19 on clinical dentistry and the relevance of the oral cavity in SARS-CoV-2 infection (Table 2) (24, 72, 93). The dental regulatory authorities quickly adapted new rules and regulations with regard to patient care and prevention of the spread of SARS-CoV-2 (72, 93). Among the health care professions, dental professionals have a high risk of making contact with diseased individuals and spreading the disease in nosocomial settings.

The American Dental Association has developed guidelines to the patient care during COVID-19 pandemic (94). The dental treatments are divided into the urgent/emergency care and routine/elective procedures. The dental emergencies which needs immediate medical attention includes life threatening conditions uncontrolled bleeding, swelling and fractures which compromise patient's airway. Urgent dental care should focus on minimizing pain, reduce or control infection, and reduce the burden on emergency departments (94). Other than that suture removal, denture adjustment, replacing fillings to alleviate pain and snipping or adjusting orthodontic appliances to prevent trauma also considered as urgent dental care. The non-urgent routine procedures include initial dental visits, routine dental cleaning and preventive therapies, aesthetic dental procedures, and extraction of asymptomatic teeth and orthodontic procedures (94). The ultimate goal is to avoid unnecessary contacts and minimize the contact to prevent the further spread of the virus in the dental care settings. The COVID-19 pandemic opens up a variety of innovative technologies for meetings such as teleconferencing, video calls, and patient photographs. Brian & Weintraub discuss use of communication media such as Teledentistry to educate and consult patients during the pandemic period, where it would greatly facilitate the prevention of unnecessary dental visits for conditions which can be temporarily alleviated at home or postponed for a later date (93). The patients with underlying health conditions such as diabetes, cancer, cardiovascular diseases (CVD) and hypertension are more susceptible to developing COVID-19 thus needs special attention. For instance, diabetes is a metabolic disorder which adversely affect the periodontal health. The periodontal disease (PD) is a chronic inflammatory disease which induce increased cytokine production and the disease severity found to be increased with COVID-19 infection (95). It is important to identify patients with underlying comorbidities and advise them on maintaining good oral hygiene to prevent the further progression of existing PD (Table 2).

Dental health care personals should be trained to be familiar with COVID-19 related signs and symptoms. The triage screening is a successful method to identify and separate out patients into three categories: (1) Triage negative (asymptomatic and negative in screening questionnaire and no fever) (2) Triage positive (positive screening questionnaire and /or fever) (3) confirmed COVID-19 cases. However, it is extremely important to take all the necessary precautions when treating the first category (82). The ability to undertake robust patient screening would facilitate the avoidance of COVID-19 transmission in dental clinics. A non-invasive rapid screening test would be of great help to identify positive cases that warrant immediate quarantine or transfer to special clinic for further treatment (88, 93). Current research are focusing on developing biomarkers for early detection, treatment and prevention of COVID-19. In the oral health care settings saliva and mucosal epithelial cells are good candidates to develop the biomarkers to identify the asymptomatic carriers (Table 2).

Given the higher viral loads in the oral cavity, it is essential to use personal protective equipment (PPE). Protective goggles or face shields, masks, gloves, and caps should be regularly worn, discarded or properly disinfected between each patient. Salivary aerosols and blood need to be protected against to reduce the risk of infection with COVID-19 (96). The use of rubber dams can significantly minimize the production of saliva-contaminated splatters, droplets and aerosols, particularly when high-speed dental hand pieces and ultrasonic devices are used. The application of a rubber dam can significantly reduce airborne particles in an ~3 foot diameter of the operational field by 70% (97). High-speed dental hand pieces without anti-retraction valves may aspirate and expel debris and fluids during dental procedures; also, the hand instruments used during general dental procedures produce a significant amount of aerosol spread (72). Good ventilation, regular and thorough surface disinfection before and after procedures with alcohol or chlorine and the proper handling of saliva-containing waste are critical in preventing the spread of COVID-19 (96). Recent studies shows that mouth rinses can reduce the SARS-CoV-2 virus load (98). Marui et al. showed that pre-procedural mouth rinses can significantly reduce microbial load in dental aerosols (99). Also, the use of pre-procedural mouth rinses before dental treatment can be advantageous during the pandemic (100).

We are too early to predict the post-pandemic effects of COVID-19. However, COVID-19 has a wide range of impacts on mental health, which can have a negative effect on the oral health of any given community; in particular, a greater impact can be seen in vulnerable populations such as people with low socioeconomic status who lack access to proper health care. On the other hand, this is a challenging time for dental health professionals and it could affect their psychological status, which can adversely affect their overall productivity. For instance, adopting new techniques to minimize the spread of disease, and reduced wages as this is associated with a decline in per capita dental visits (93). The timely vaccination of health care professionals and the vulnerable population is now a strategic priority for the prevention of COVID-19 in many countries. Dai and Gao in their progressive article discussed about the different vaccine candidates against SARS-CoV-2 (i.e., Inactivated virus vaccines, virus like particle or nanoparticle viruses, protein subunit vaccines, virus-vectored vaccines, DNA and mRNA vaccines and live attenuated vaccines) and compare their effectiveness against COVID-19 (101). The world largest vaccination campaign begins with the BioNTech/Pfizer, and Moderna/NIAID vaccines and Oxford-AstraZenca's is now authorized and added to this mass prevention battle against COVID-19. However, the immunization programs needs further investigations for their effectiveness against the novel variants of SARS-CoV-2 (101, 102). Further studies need to be performed to identify the pathogenicity of SARS-CoV-2 on specific epithelial organs of the oral cavity and its effect on oral health. Salivary research can be directed toward designing rapid identification test kits as a chair-side test prior to any dental procedures in order to diagnose SARS-CoV-2 carriers. The use of corticosteroids, antivirals and antibiotics to treat the oral mucosal lesions of COVID-19 needs to be studied further using large samples in different demographic settings. The ongoing COVID-19 pandemic is an eye-opener to all of mankind to be vigilant and prepared to fight future pandemics. Specifically, scientific knowledge gained from this pandemic can be useful in designing public healthcare protocols to prevent future pandemics and vaccines, and therapeutic treatment research will be invaluable in patient management in any virulent coronavirus infections.

## Author Contributions

DSA and RKR contributed to the conception and critically revised the manuscript. Both authors contributed to the article and approved the submitted version.

## Conflict of Interest

The authors declare that the research was conducted in the absence of any commercial or financial relationships that could be construed as a potential conflict of interest.
